# QMEANclust: estimation of protein model quality by combining a composite scoring function with structural density information

**DOI:** 10.1186/1472-6807-9-35

**Published:** 2009-05-20

**Authors:** Pascal Benkert, Torsten Schwede, Silvio CE Tosatto

**Affiliations:** 1 Swiss Institute of Bioinformatics, Biozentrum, University of Basel, Klingelbergstrasse 50/70, 4056 Basel, Switzerland; 2 Department of Biology, Universita' di Padova, Viale G. Colombo, 35121 Padova, Italy

## Abstract

**Background:**

The selection of the most accurate protein model from a set of alternatives is a crucial step in protein structure prediction both in template-based and *ab initio *approaches. Scoring functions have been developed which can either return a quality estimate for a single model or derive a score from the information contained in the ensemble of models for a given sequence. Local structural features occurring more frequently in the ensemble have a greater probability of being correct. Within the context of the CASP experiment, these so called consensus methods have been shown to perform considerably better in selecting good candidate models, but tend to fail if the best models are far from the dominant structural cluster. In this paper we show that model selection can be improved if both approaches are combined by pre-filtering the models used during the calculation of the structural consensus.

**Results:**

Our recently published QMEAN composite scoring function has been improved by including an all-atom interaction potential term. The preliminary model ranking based on the new QMEAN score is used to select a subset of reliable models against which the structural consensus score is calculated. This scoring function called QMEANclust achieves a correlation coefficient of predicted quality score and GDT_TS of 0.9 averaged over the 98 CASP7 targets and perform significantly better in selecting good models from the ensemble of server models than any other groups participating in the quality estimation category of CASP7. Both scoring functions are also benchmarked on the MOULDER test set consisting of 20 target proteins each with 300 alternatives models generated by MODELLER. QMEAN outperforms all other tested scoring functions operating on individual models, while the consensus method QMEANclust only works properly on decoy sets containing a certain fraction of near-native conformations. We also present a local version of QMEAN for the per-residue estimation of model quality (QMEANlocal) and compare it to a new local consensus-based approach.

**Conclusion:**

Improved model selection is obtained by using a composite scoring function operating on single models in order to enrich higher quality models which are subsequently used to calculate the structural consensus. The performance of consensus-based methods such as QMEANclust highly depends on the composition and quality of the model ensemble to be analysed. Therefore, performance estimates for consensus methods based on large meta-datasets (e.g. CASP) might overrate their applicability in more realistic modelling situations with smaller sets of models based on individual methods.

## Background

Generally, protein structure prediction consists of a conformational sampling step followed by a scoring step in which the best model is selected from the ensemble. The relative importance of the two steps depends on the modelling difficulty and the details of the specific method. In the conformational sampling step of *ab initio *structure prediction methods it is common practice to generate a vast number of models and to subsequently select the best candidates based on an energy function [1,2]. Until several years ago, in comparative modelling usually only a few, if any, alternative models have been generated and the quality of the prediction was rarely better than the best template. However, in recent years there has been a clear trend in the field to generate a variety of models based on different template structures (or combinations thereof) and/or alternative alignments and to select the best candidate based on the estimated quality of the resulting models [3-10]. In order to cope with the uncertainties in modelling, early decision making, such as choosing the best template or alignment, can be postponed and performed at a later stage in the modelling pipeline based on the quality of the resulting structural model. For this last step, scoring functions for selecting the highest quality model among alternatives are of crucial importance.

These scoring functions fall into one of two categories, namely consensus or clustering methods which rely on the analysis of the structural density in the ensemble of models and approaches being able to estimate the quality of a single model without relying on consensus information. The basic idea of consensus-based methods is that conformations predicted more frequently are more likely to be correct than structural patterns occurring in only a few models [11-15]. The second category includes methods taking into account evolutionary information [16-18], stereochemical plausibility of the models [19,20] and the environment compatibility of their residues [21] as well as energy-based methods which include physics-based energy functions [22,23] and knowledge-based statistical potentials [24-29]. Composite scoring functions analysing multiple structural features have been introduced and shown to perform better than any single term [30-35].

Quality estimation can be performed on different dimensions: relative vs. absolute and global vs. local. The estimation of the relative quality of a model compared to a set of alternatives is, as mentioned above, a fundamental step in protein structure prediction and also in optimisation techniques (*i.e*. refinement). On the other hand, the estimation of the absolute quality of a model is of tremendous importance for the biological community since it is the quality of the model which dictates its biological applicability (*e.g*. for mutagenesis studies, virtual screening and molecular replacement) [36-38]. Traditionally, scoring functions have been assessed with regard to their ability to rank models by quality, while the estimation of absolute values of model quality has been only marginally addressed in the literature. Besides the global quality, local error estimation on a per residue basis has become an active field of research [17,39]. Although the accuracy of local predictions is limited, these methods may be very valuable for biologists by helping them to discriminate between reliable and unreliable regions in the model.

Model quality assessment programs have been evaluated for the first time in a community-wide experiment in 2004 as part of Critical Assessment of Fully Automated Structure Prediction (CAFASP) [40] and most recently at CASP7 [41,42]. The assessment of the predictions submitted to the quality assessment category of CASP7 clearly indicates that consensus based methods such as Pcons [12] outperform current scoring functions operating on single models. On the other hand, methods relying solely on structural density information have inherent limitations: First, they are not able to provide an estimate of the absolute quality of a single model or to rank just a small set of models. Second, these methods tend to fail when the highest quality candidates are far away from the dominant structural cluster of the ensemble. Outstanding predictions which are far removed from the bulk of the remaining models are hardly recognised [43,44], and, in the case of hard free modelling targets, the ensemble does often not contain any meaningful density information at all. The approach pursued by Lee and co-workers [45] for the quality assessment category of CASP7 was also quite successful. This group produced quite accurate models for the template-based modelling category [43] and defined the quality of all other models as relative distance to their own models.

Based on these findings, we present in this paper a new approach to model quality estimation which combines different aspects of the approaches described above while simultaneously minimising their weaknesses. We use an optimised version of our recently published composite scoring function QMEAN [33] in order to define an ensemble of reference models which is used to calculate the structural consensus score. This method, called QMEANclust, combines a scoring function able to assess single models and perform an initial ranking with the strengths of using structural density information. Due to the pre-selection step, QMEANclust represents a compromise between the rigorous clustering strategy of Pcons (comparison to all models) and the strategy to define quality by comparison to a "best reference model". Based on the model ranking of QMEANclust, it is investigated whether using the ensemble of models for a given target sequence to retrieve target-specific statistical potentials [14] can lead to a further performance improvement (selfQMEAN).

The paper is structured as follows: First we describe the optimised QMEAN scoring function. We demonstrate that the inclusion of an all-atom interaction term in addition to the residue-level term improves the performance both with respect to correlation between predicted model score and degree of nativeness and in the task of selecting the best model. Then we compare different strategies to combine QMEAN with structural density information resulting in two versions of QMEANclust as well as in the selfQMEAN scoring function. We show that QMEANclust is indeed able to counteract the inherent limitations of purely consensus-based methods. All three scoring functions are compared to state-of-the-art methods on the basis of two comprehensive test sets. Finally, local versions of the three scoring functions for the per-residues error estimation are presented and the performance is compared to a recently published method.

## Results and Discussion

### QMEAN: Composite scoring functions for the evaluation of single models

We recently described the QMEAN composite scoring function consisting of a linear combination of five terms including 3 statistical potentials [33]. The combination of broadly orthogonal information has been shown to improve model selection. The QMEAN composite scoring function includes a torsion angle potential over three consecutive amino acids for the analysis of the local geometry of a model, a solvation potential describing the burial status of the residues and a distance-dependent interaction potentials based on Cβ atoms for the assessment long-range interactions. Two terms describing the agreement of predicted and calculated secondary structure and solvent accessibility are also included. In this work, the QMEAN composite scoring function has been extended by an all-atom distance-dependent interaction potential term in order to capture more structural detail. A short description of all QMEAN versions and the terms used in their calculation can be found in Table 1.

**Table 1:** Short description of the terms and their combinations used in this work.

**scoring function**	**Description**
torsion	Extended torsion potential over 3 consecutive residues. Bin sizes: 45 degree for the centre residue, 90 degree for the 2 adjacent residues
pair residue	Residue-level, secondary structure specific interaction potential using Cβ atoms as interaction centres. Range 3...25 Å, step size: 1 Å
solvation	Potential reflecting the propensity of a certain amino acid for a certain degree of solvent exposure based on the number of Cβ atoms within a sphere of 9 Å around the centre Cβ.
pair all-atom	All-atom, secondary structure specific interaction potential using all 167 atom types. Range 3...20 Å, step size: 0.5 Å
SSE agreement	Agreement between the predicted secondary structure of the target sequence (using PSIPRED) and the calculated secondary structure of the model (using DSSP).
ACC agreement	Agreement between the predicted relative solvent accessibility using ACCpro (buried/exposed) and the relative solvent accessibility derived from DSSP (> 25% accessibility => exposed)
QMEAN3	linear combination of torsion, pair residue, salvation
QMEAN4	linear combination of torsion, pair residue, solvation, pair all-atom
QMEAN5	linear combination torsion, pair residue, solvation, SSE, ACC
QMEAN6	linear combination of torsion, pair residue, solvation, pair all-atom, SSE, ACC

The first section of Table 2 shows the target-averaged performance of different QMEAN versions on the CASP7 dataset consisting of all server models submitted for 98 targets. The other sections show the performance of various QMEANclust and selfQMEAN implementations which, in contrast to QMEAN, take into account consensus information. The weighting factors for the different composite scoring functions are optimised on the CASP6 training set.

**Table 2:** Comparison between QMEAN, various QMEANclust implementations and selfQMEAN on all CASP7 server models.

**QMEAN implementation**	**Pearson**	**Spearman**	**Sum(GDT)**
***QMEAN:***			
QMEAN3	0.645	0.551	50.17
QMEAN3 * fraction modelled	0.663	0.605	51.92
QMEAN4	0.647	0.540	49.57
QMEAN4 * fraction modelled	0.671	0.609	52.65
QMEAN5	0.729	0.630	54.87
QMEAN5 * fraction modelled	0.740	0.676	55.32
QMEAN6	0.741	0.638	56.36
QMEAN6 * fraction modelled	0.752	0.684	**56.70**

***QMEANclust: no preselection***			
Median	0.872	0.812	56.64
Mean *(~3D-jury based on GDT_TS)*	0.889	0.821	57.16
Weighted mean	0.883	0.824	57.63

***QMEANclust: QMEAN Z-score > x***			
Median: Z-score > -1	0.877	0.815	57.05
Mean: Z-score > -1	0.876	0.817	57.30
Weighted mean: Z-score > -1	0.882	0.823	57.60
Median: Z-score > 0	0.884	0.824	57.52
Mean: Z-score > 0	0.879	0.822	57.35
Weighted mean: Z-score > 0	0.882	0.826	57.31
Median: Z-score > 0.5	0.885	0.828	57.33
Mean: Z-score > 0.5	0.880	0.830	56.96
Weighted mean: Z-score > 0.5	0.883	0.832	57.18

***QMEANclust: top × percent models***			
Median: 20% TBM, 20% FM	0.888	0.842	57.37
Median: 10% TBM, 10% FM	0.890	**0.844**	57.83
Median: 5% TBM, 5% FM	0.873	0.826	56.98
Median: 10% TBM, 20% FM	0.886	**0.844**	57.23
Median: 20% TBM, 10% FM	**0.892**	0.842	57.97

***QMEANclust: ΔQMEAN-score from max***			
Median: Δ < 0.05 Å TBM, Δ < 0.05 Å FM	0.867	0.826	57.65
Median: Δ < 0.1 Å TBM, Δ < 0.1 Å FM	**0.892**	0.837	57.69
Median: Δ < 0.05 Å TBM, Δ < 0.1 Å FM	**0.892**	0.841	**58.11**
Median: Δ < 0.1 Å TBM, Δ < 0.05 Å FM	0.868	0.822	57.23

***selfQMEAN:***			
Linear combination of 5 terms (w/o all-atom)	0.811	0.755	55.53
Sum of Z-scores (5 terms)	0.830	0.749	**56.60**
Sum of Z-scores (6 terms)	**0.832**	0.753	55.60

Average correlation coefficient and total maximum GDT_TS score of the selected models of different QMEAN versions obtained on the test set containing all CASP7 server models. A description of all QMEAN versions is given in Table 1. For the QMEANclust consensus score, a multitude of strategies for pre-selecting reference models based on QMEAN score is investigated. The models of the reference set are defined based on a certain Z-score cut-off, by using only a percentage of top scoring models or by including only models being close to the highest scoring model. The different cut-offs used for template-based modelling targets (TBM) of free modelling targets (FM) are indicated. Underlined values are used in Table 3 for comparison to other methods. The selfQMEAN scoring function is based on ensemble-specific statistical potentials.

For each QMEAN version, the performance of an alternative implementation which penalises incomplete models by multiplying the score by the fraction of modelled residues is given as well. Taking into account the coverage of the models with respect to the target sequence considerably improves the correlation to the GDT_TS score [46] by penalising incomplete models with otherwise good stereochemistry. This performance increase in estimating the relative model quality can be attributed to the fact that the GDT_TS score, traditionally used in the assessment of CASP, is by definition dependent on model completeness. Table 2 underlines that a large increase in performance can be obtained by including predicted secondary structure and solvent accessibility agreement terms as shown previously (QMEAN3 vs. QMEAN5 and QMEAN4 vs. QMEAN6). The integration of an all-atom term (QMEAN5 vs. QMEAN6 in Table 2) further improves the correlation between predicted quality of the model and its similarity to the native structure. More importantly, the all-atom term increases the ability of the scoring function to select good models. This is reflected by the significantly higher (p-value = 0.03 in a paired t-test) total GDT_TS score of the best models selected by QMEAN6 of 56.70 compared to 55.32 for QMEAN5.

For comparison, the performance of the top methods of the quality assessment category of CASP7 are shown in Table 3 together with the maximum GDT_TS of the top performing server, *i.e*. a scoring function that always selects the models of the Zhang server [43,47]. For a description of the other methods visit the CASP7 website . The GDT_TS values as well as the data of the other methods are based on the quality assessment data of CASP7 and the data of TASSER-QA have been kindly provided by the authors [35].

**Table 3:** Comparison of the best QMEAN versions with other methods participating in CASP7.

**Scoring function**	**Pearson**	**Spearman**	**sum(GDT)**
QMEAN	0.752	0.684	56.70
Circle-QA	0.718	0.643	56.03
ProQ	0.700	0.571	54.29
ProQlocal	0.698	0.563	54.17
Bilab	0.683	0.561	54.50
ModFOLD	0.661	0.580	54.19
ABIpro	0.653	0.605	56.40

selfQMEAN	0.830	0.749	56.60
QMEANclust	**0.892**	**0.841**	**58.11**
Pcons	0.801	0.714	54.36
TASSER-QA	0.828	0.785	57.23

Zhang server	-	-	57.35
Random model selection	-	-	*46.19*
Best model per target	-	-	*62.00*

Average correlation coefficient and total maximum GDT_TS score of the optimised QMEAN, QMEANclust and selfQMEAN versions and the top performing methods of CASP7. Only scoring functions with predictions for all 98 targets are shown.

**Figure 1:**
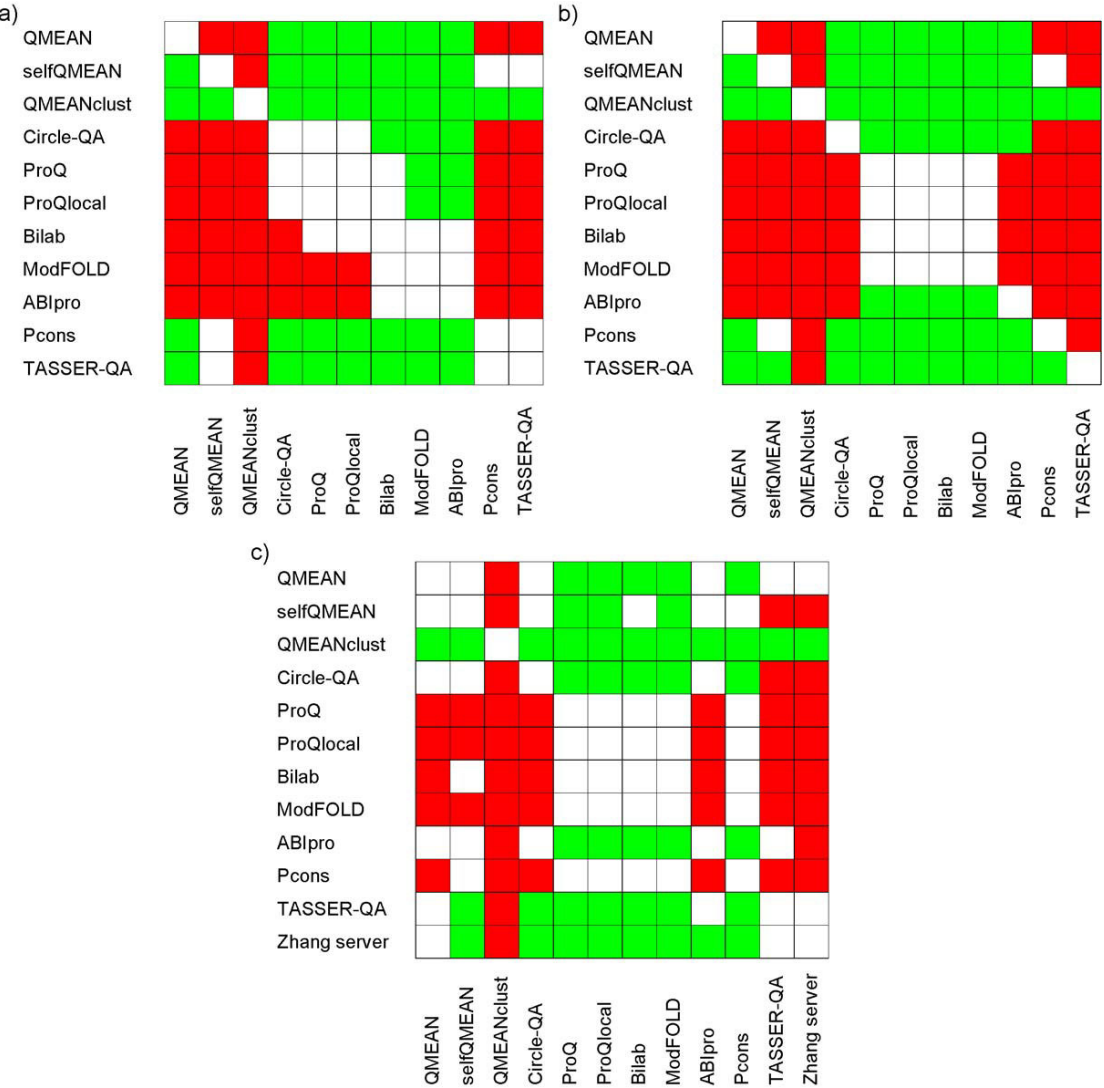
**Analysis of the statistical significance based on a one-sided paired t-test (95% confidence level)**. Green: Method denoted on the horizontal performs significantly better. Red: Method denoted on the horizontal performs significantly worse. a) Pearson's correlation coefficient, b) Spearman's rank correlation coefficient, c) GDT_TS values of the models selected model by a scoring function.

A statistical analysis of the above results is given in Figure 1. From the scoring functions being able to return a score for a single model, QMEAN6 shows the best correlation coefficient (both Pearson and Spearman) over all methods participating in CASP7 (Table 3, first section). The difference is statistically significant at the 95% confidence level based on a paired t-test. QMEAN also shows the best performance in selection of good models for each target as reflected by the highest total GDT_TS values followed by ABIpro and Circle-QA, but in this case the difference is statistically not significant. Scoring functions which take into account structural density information such as selfQMEAN and QMEANclust produce considerable higher correlation coefficients and total GDT_TS scores (see below).

A further improvement may be achieved by using more specialised QMEAN versions for different modelling situations, such as QMEAN with all-atom term for template based targets and without for free modelling targets. First results suggest that the overall effect is only marginal and that the QMEAN version including the all-atom term leads to a better performance over the whole difficulty range. Using one scoring function for all modelling situations is not ideal as highlighted recently by Kihara co-workers [48]. They showed that for a threading scoring function consisting of two terms, different weighting factor combinations are optimal for different protein families. Therefore, training weighting factors specifically for proteins of similar size and amino acid or secondary structure composition may improve the performance, especially in the prediction of absolute values of model quality [49]. Optimising weighting factors in composite scoring functions based on a linear combination of terms is complicated by the fact that the different terms are dependent on the protein size which influences to ability of the combined scoring function to predict the absolute quality.

### QMEANclust: including structural density of the model ensemble

In this section we describe a new method, termed QMEANclust, which combines the QMEAN scoring function with structural density information derived from the ensemble of models. In the straightforward implementation of methods based on structural density information, the score for a given model is calculated as its average (or median) distance to all other models in the ensemble. Different similarity measures are used for building the distance matrix: *e.g*. MaxSub [50] in 3Djury [11], LGscore [51] in Pcons [12] and TMscore [52] in the consensus method described in MODfold [53]. In this work, the GDT_TS score [46], a well established similarity measure in the CASP assessment, is used. In all the above mentioned implementations, the single models are equally weighted in the calculation of the final score, no matter how good or bad a model is. In 3Djury only model pairs above a certain distance cut-off are considered in the calculation.

Clustering methods tend to fail when the top models are far away from the most prominent structural cluster or when there is no structural redundancy present in the ensemble that can be captured. Especially for difficult, template-free modelling targets the best models are usually not the most frequent conformations in the ensemble (at least not in the CASP decoy sets). In order to cope with the limitations of current clustering approaches, we investigated two strategies for the combination of the QMEAN composite scoring function and structural density information from the ensemble. In the first approach, QMEAN is used to select a subset of higher quality models against which the subsequent distance calculations are performed. The final score for a given model is defined as the median distance of this model to all models in the subset (strategy denoted as *median *in Table 2). An implementation based on the mean instead of the median GDT_TS is also investigated. In the second approach, the models are weighted according to their QMEAN score (denoted *weighted mean*); For deriving the distance matrix, the distance of a given model to more reliable models (*i.e*. to models having better QMEAN scores) is weighted stronger, which in turn reduced the influence of random models on the calculation.

Different strategies and cut-offs for model selection have been investigated. A benchmark of several alternative implementations on the CASP7 test set can be found in Table 2. In comparison to the performance of QMEAN, considerably higher correlation coefficients are obtained for all QMEANclust versions (r = 0.752 vs. r = 0.892).

If the whole ensemble of models is used in the derivation of the distance matrix (no pre-selection), the weighted mean performs comparable or better than taking the mean or median both in terms of correlation between predicted and observed model quality and the ability to identify good models. If only a subset of high-quality models is used in the calculation of the distance matrix, a score based on the distance median produced the best results and is used in the final version. Three different approaches have been investigated in order to select a subset of models based on QMEAN: (1) selection based on the Z-scores which are calculated by subtracting from each model the mean QMEAN score of the ensemble and dividing it by its standard deviation, (2) selection of a certain percentage of top ranking models as well as (3) a strategy in which only models with a similar QMEAN score as the top ranked model are used in order to cope with qualitatively outstanding predictions.

A combination of both pre-selection of models based on QMEAN and weighting the distances according to QMEAN in the subsequent clustering calculations is not useful as shown for the selection based on Z-scores. Z-scores have been calculated based on the model QMEAN score and only models above a given Z-score threshold are used for the clustering process. Table 2 shows that, with increasing Z-score threshold (*i.e*. fewer models from the ensemble are used in distance calculations), the ability of the *weighted mean *strategy to select good models gradually decreases, whereas the performance of the *median *strategy increases (until Z-score > 0). Using the median rather than the mean reduces the influence of outliers in smaller data sets. For the other two selection strategies, only *median *is shown, *i.e*. the final QMEANclust score of a model is the median distance of this model to all other models in the subset selected by the given strategy.

Model selection based on Z-scores has several disadvantages: the number of models selected using a given Z-score cut-off is highly dependent on the modelling difficulty. For an easy template based modelling target, the models in the ensemble tend to be very similar and there are no models with high Z-scores (*e.g*. for some targets there are no models with a Z-score greater than 1). On the other hand, for free modelling targets there are sometimes outstanding predictions compared to the bulk of more or less random models. Capturing these predictions in the selection step is the only way to circumvent the inherent limitations of consensus based methods. Furthermore, different selection cut-offs may be needed for template based modelling targets (TBM) and free modelling targets (FM) since the former contain much more structural redundancy which can be captured by clustering methods and more targets can potentially be used in the calculation of the distance matrix.

In the fourth section of Table 2, the results of a selection strategy based on a fixed percentage of top scoring models are shown. A total GDT_TS of 57.97 is achieved by using the top 20% models for TBM targets and top 10% for FM targets. Discrimination between TBM and FM targets is done based on mean QMEAN score by assigning targets with a model averaged QMEAN score above 0.4 to the template-based modelling category. This cut-off has been derived empirically by comparing the score distributions of FM and TBM targets (data not shown). The better performance of the approach, which uses a more tolerant model selection for TBM targets, may be attributed to the fact that the model ensemble of TBM targets contains more useful consensus information. In the case of FM targets, QMEAN is often able to identify some of the better models which are subsequently used in the consensus calculation.

Alternatively, a simple selection strategy aiming at capturing outstanding predictions has been investigated (fifth section of Table 2). Only models with a similar QMEAN score compared to the highest scoring model are considered for the distance calculation. A selection of models within 0.05 QMEAN units from the maximum for TBM targets and 0.1 units for FM targets results in a total GDT_TS of 58.11. Since the TBM models are structurally more homogenous, more models are selected in TBM targets than FM targets using these thresholds. For the subsequent comparison to other methods, the best versions of QMEAN, QMEANclust and selfQMEAN (see below) are used. The corresponding values are underlined in Table 2.

At CASP7, none of the quality assessment programs (clustering and non-clustering methods) was able to select better models out of the ensemble of server models than the Zhang server [54] submitted for each target [35,41,44]. The best QMEANclust implementation shows a better model selection performance than TASSER-QA [35] and a naive scoring function that simply takes the Zhang server models (total GDT_TS of 58.11 vs. 57.35). The difference is statistically significant at the 95% confidence level based on a paired t-test. Figure 1 underlines that QMEANclust and the single model scoring function QMEAN show a statistically better (p = 1.9*10^-5 ^and p = 0.009, respectively) selection performance than Pcons, the best performing clustering based method at CASP7. In terms of correlation between predicted model quality and degree of nativeness, QMEANclust has significantly higher Pearson's (0.892 vs. 0.828 of TASSER-QA) and Spearman's (0.841 vs. 0.785) correlation coefficients than TASSER-QA and any other tested scoring function.

Although the ability of QMEANclust to pick the best model is better than a naive predictor that simply picks Zhang models, it can still potentially be improved. The weighting factors for the QMEAN scoring function used for model prioritisation has been optimised for regression and not for selecting the best model. Qui *et al*. [34] recently described an approach in which a composite scoring function has been optimised for model selection using support vector machines. Most current scoring functions ignore a trivial parameter for the estimation of model quality: the presence and closeness of a structural template which can be used to build the model [55]. Zhou and Skolnick [35] recently described a scoring function in which the extent a model is covered by fragments of templates identified by threading is used as quality measure. QMEAN could benefit of such a term representing orthogonal information to the present implementation.

### selfQMEAN: use of statistical potential terms derived from model ensemble

The idea of using the ensemble of models for a given target as basis to derive target-specific statistical potential terms has previously been investigated [14]. In their work, Wang *et al*. generated a decoy-dependent implementation of the RAPDF interaction potential [56] by deriving the distance frequencies from the models in the decoy set and weighting each count according to the RAPDF score of the model. This decoy-dependent statistical potential performed better that the original RAPDF scoring function but not as good as a simple density score based on the average RMSD of a model to all others. Here we followed a similar strategy with the difference that a combined scoring function using multiple statistical potentials is used and that an improved density scoring function (QMEANclust) is used for weighting the models contributing to the selfQMEAN score (see Methods). As can be seen from Table 2, while selfQMEAN generates considerably higher correlation coefficients than QMEAN, the ability to select good models does not improve. The decoy-dependent scoring function does not perform better than QMEANclust, which is based on structural density information alone. Building a composite scoring function based on target-specific potentials is problematic since the weighting factors are highly dependent on the modelling difficulty: Ensembles containing lots of very similar models, *e.g*. in high accuracy template based models, result in much lower absolute energies in the statistical potential terms than sets of diverse models. We tried to circumvent the problem by just adding the energy Z-scores of each term. These results suggest that the level of detail captured by target-specific scoring functions decreases compared to the direct derivation of structural differences based on consensus methods. The structural density information seems to be captured more precisely when directly derived from the distance matrices without doing the detour using model ensemble specific statistical potentials. These methods are also not able to overcome the limitations of purely consensus based methods being determined by the most dominated structural cluster.

### Comparison of QMEANclust with 3Djury-like consensus method

**Figure 2:**
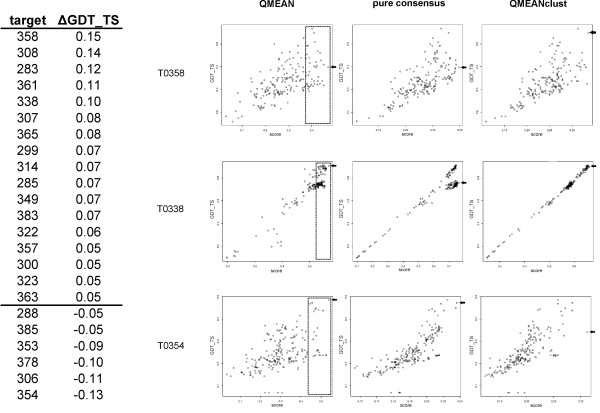
**Comparison of QMEAN, a 3d-Jury like approach and QMEANclust on 3 selected CASP7 targets**. The table shows the GDT_TS difference between the best select model by QMEANclust and the 3D-jury approach. Correlations between predicted score and GDT_TS of three targets are shown for QMEAN, 3D-jury and QMEANclust (from left to right). The dashed areas mark the models selected by QMEAN as the basis for QMEANclust. The arrow on the right of each plot denotes the best selected model.

In this section we address the question whether QMEANclust and its strategy of selecting a subset of high quality models for the calculation of the structural density is really superior to pure consensus methods and whether the new method is able to identify good models even if they are far away from the most dominant structural cluster. For the comparison we use a 3D-jury like [11] implementation based on GDT_TS (*i.e*. the score of a model is simply its *mean *GDT_TS to all other models of a given target). As can be seen from Table 2, this approach achieves a total GDT_TS of 57.16 compared to 58.11 of QMEANclust. A closer inspection of the performance differences on the 98 CASP7 targets reveals that QMEANclust in many cases is able to circumvent the inherent limitations of 3D-jury. The table on the left-hand side of Figure 2 lists all targets where the model selection based on QMEANclust is at least 0.05 GDT_TS units better (17 targets) or worse (6 targets) than the one based on 3D-jury. The results of three targets are shown in more detail in Figure 2. Two examples are shown (T0358, T0338) in which the pre-selection of models based on QMEAN (dashed area on plots in the first column) resulted in better model selection by QMEANclust compared to 3D-jury. The results are especially pronounced in the case of target T0308. The models of this target seem to be based on two categories of templates and the majority of groups seem to have used the less appropriate one. The dashed area containing all models within a QMEAN score of 0.05 units from the best ranked model captures vast majority of the models of the highest quality cluster and only a fraction of the dominant structural cluster. The pre-selection step results in a QMEANclust ranking which is not dominated by the models of the second cluster as opposed to the 3D-jury ranking. The correlation coefficients are 0.923 for QMEAN, 0.931 for the 3D-jury like approach and 0.997 for QMEANclust.

Targets T0354 represents an example in which QMEANclust failed to improve over a purely clustering based approach. This can be attributed to the inconsistencies in the QMEAN ranking in which a set of similar but very poor models have been ranked too high. For this target the best model selection would have been actually obtained by QMEAN (as denoted by the arrow on the right).

### MOULDER test set: Performance in a realistic modelling situation

As the QMEAN scoring function has been optimised on CASP6 models and tested on CASP7 models, one might raise the argument that it tends to be over-trained for this special situation and also to the GDT_TS score used there. Therefore we analysed the performance of QMEAN on the MOULDER test set which represents a more realistic modelling situation. The MOULDER test set consists of 20 different targets, each with 300 alternative models generated by MODELLER [57].

Table 4 shows a comparison between QMEAN and its components and several well-established scoring functions recently benchmarked by Eramian *et al*. [32]. The RMSD difference (in Ångstrom) between the best model in the ensemble and the one selected by the scoring function is given averaged over all targets. As in the original paper, for each target, the calculations are repeated 2000 times with a random subset (25%) of models in order to increase the robustness of the statistics. A description of the terms not explained here can be found in the in the paper by Eramian *et al*. They investigated a total of 40 terms and built a composite scoring function combining the 10 best performing terms using support vector machines *(SVM_SCORE)*. Table 4 highlights the strength of QMEAN (especially QMEAN6 including the all-atom term) in model selection. Although no machine learning algorithm has been used to combine the terms, QMEAN performs better than the SVM approach. This can be at least partly attributed to the secondary structure specific all-atom distance-dependent interaction potential. The use of a secondary structure specific version compared to the standard implementation leads to consistently better results on the CASP6 and CASP7 test set as well as on the MOULDER set (data not shown). On the MOULDER data set, the all-atom term of QMEAN performs better than the well-established DFIRE and DOPE scoring functions as well as the ROSETTA score. The torsion angle potential term implemented in QMEAN shows a very poor performance on this test set. The torsion angle distribution in the decoy structures is possibly too similar to be useful for model discrimination based on the very coarse-grained torsion angle potential over three residues. But this term has been shown to be very helpful in other test sets and especially in the task of recognising the native structure [33].

**Table 4:** Performance comparison of QMEAN to other single model scoring functions based on the MOULDER test set.

**Scoring function**	**Mean ΔRMSD [Å]**	**Std. dev. [Å]**
torsion	4.50	4.06
pairwise Cbeta, SSE	1.48	3.00
Salvation Cbeta	1.06	1.91
SSE agreement	0.92	1.24
ACC_agreement	0.79	1.07
pairwise all-atom, SSE	0.68	0.96
QMEAN5	0.42	0.59
**QMEAN6**	**0.40**	**0.59**

SIFT	5.68	5.20
Anolea_Z	1.94	2.29
SOLVX	1.76	2.21
Xd	1.68	2.63
FRST	1.55	2.41
MP_SURF	1.36	1.90
MP_PAIR	1.20	1.70
EEF1	1.09	1.52
GB	1.06	1.36
DOPE_BB	0.96	1.27
PROSA_COMB	0.89	1.52
GA341	0.84	0.86
MODCHECK	0.83	1.29
MP_COMBI	0.82	1.19
DFIRE	0.81	1.37
DOPE_AA	0.77	1.21
ROSETTA	0.71	1.05
SVM_SCORE	0.46	0.66

The table shows the RMSD difference (in Ångstrom) between the selected model by the scoring function and best model in the ensemble, averaged over the 20 protein targets of the MOULDER test set. In order to increase the robustness of the statistics, each calculation is repeated 2000 times on random subsets of 25% of the model ensemble. For comparison, the mean ΔRMSD and standard deviations for QMEANclust (based on consensus scoring of all 300 models) are 1.15 and 1.39 Å respectively. For a detailed comparison of QMEAN and QMEANclust see Table 5.

The performance of QMEANclust on the MOULDER test set is highly dependent on the composition and quality of the decoy set as is apparent from data in Table 5. The data are sorted by increasing median RMSD of the 20 decoy sets and no re-sampling has been applied such that the entire set of 300 models is used per target. The performance of QMEANclust decreases with increasing diversity of the decoy set which is also reflected by number of near-native models in the set. QMEANclust shows a considerably worse model selection performance compared to QMEAN on the decoy sets in the lower part of the table. On the 8 decoy sets with less then 50 near-native models (*i.e*. models below 5 Å), the difference is statistically significant in a paired t-test (p-value 0.05). These model ensembles do not seem to contain useful structural density information which could be captured since only few models have a RMSD below 5 Å. On the entire MOULDER test set the QMEAN scoring function achieves an average ΔRMSD of 0.57 Å compared to 1.15 Å of QMEANclust. Overall, the single model scoring function QMEAN selects for 4 targets the best available model in the ensemble and for 17 targets a model deviating less than 1 Å. On the other hand, QMEANclust performs equally well on decoy sets populated with a high fraction of near-native models. The average ΔRMSD over the 12 targets containing at least 50 near-native models of QMEAN is 0.58 Å compared to 0.46 Å for the consensus method QMEANclust. The performance difference is statistically not significant (p-value of 0.55 in a paired t-test). Although the results have been obtained on a small test set of only 20 targets, they underline the fact that the performance of consensus scoring functions is highly dependent on the composition of the model ensemble to be analysed.

**Table 5:** Comparison between QMEAN and QMEANclust in the task of selecting near native models on the MOULDER test set.

			**ΔRMSD [Å]**	
			
**targets**	**median RMSD [Å]**	**# < 5Å**	**QMEAN**	**QMEANclust**
2cmd	5.76	100	2.75	0.67
1bbh	6.49	86	0.00	0.17
2mta	6.66	119	0.29	0.31
1dxt	7.19	79	1.11	0.72
2pna	7.29	57	0.14	0.14
1lga	8.17	106	0.82	1.10
1mup	8.18	65	0.40	0.36
8i1b	8.34	115	0.62	0.47
2afn	8.54	42	0.12	0.58
2fbj	8.84	59	0.29	0.26
1mdc	9.27	105	0.07	0.18
1onc	10.46	106	0.47	0.15
1c2r	10.46	7	0.00	1.95
2sim	10.98	55	0.00	0.96
1cid	11.16	0	0.11	0.63
1gky	11.56	15	0.66	1.16
1cau	11.92	11	0.42	3.54
1eaf	12.64	1	0.34	1.72
1cew	14.74	21	2.77	2.24
4sbv	17.40	1	0.00	5.74

*average*	*9.80*	*57.5*	*0.57*	*1.15*

The first two data columns contain the median RMSD of the models in the decoy set and the number of models with RMSD < 5 Å (out of totally 300). For all 20 target proteins, the RMSD difference (in Ångstrom) is given between the selected model and best model in the ensemble.

### QMEANlocal: local quality estimation

Structural density information can not only be used globally by comparing entire models but also on the residue level by analysing the local structural diversity among the models [44]. A region modelled entirely different in one model compared to the majority of the others is very unlikely to be correct. Table 6 shows a comparison of clustering and non-clustering approaches concerning local quality estimation on the CASP7 test set.

**Table 6:** Comparison of consensus and non-consensus based methods in the estimation of the local model quality.

**Scoring function**	**r**	**tau**	**ROC_avg_**	**ROC_all_**	**low_10%_**	**top_10%_**
QMEANclust_local	**0.83**	**0.53**	**0.88**	**0.93**	2.2	**29.5**
selfQMEAN_local	0.49	0.35	0.84	0.90	1.3	5.8
QMEAN_local	0.43	0.32	0.80	0.83	**0.8**	4.3
ProQres	0.28	0.26	0.74	0.77	0.9	5.8

r = average Pearson's correlation coefficient; tau = Kendalls's tau on a per model basis; ROC = area under ROC curve averaged over all 98 targets (avg) or using all residues pooled together (all); low/top10% = average Cα distance of the 10% lowest/highest scoring residues per target.

The per-residue predictions based on QMEAN, QMEANclust and selfQMEAN are compared to the recently published ProQres scoring function (non-consensus method). In ProQres a neural network is used to combine several local descriptors [17]. Recently, Fasnacht *et al*. [39] published a local composite scoring function based on different terms combined by support vector machines resulting in a slightly better performance. The SVM approach, as well as ProQres, have been shown to outperform classical scoring functions such as Verify3D [21] and ProsaII [58]. A direct comparison to these methods is therefore not necessary and a rigorous benchmark against other local quality estimation methods is beyond the scope of this work. Rather, the general performance differences of non-clustering, clustering and "self-clustering" methods should be highlighted and discussed here.

The QMEANlocal composite scoring function described here consists of a linear combination of 8 structural descriptors. The local scores are calculated over a sliding window of 9 residues which resulted in the best performance compared to alternative window sizes (data not shown). In analogy to the global QMEAN version, 4 statistical potential terms are combined with 2 terms describing the local agreement between predicted and measured secondary structure and solvent accessibility. Additionally, two trivial descriptors are used: the average solvent accessibility and the fraction of residues in the segment with no defined secondary structure. The weighting factors have been optimised on the models submitted to CASP6 with the Cα distance as target function (see Methods for details).

QMEANlocal estimates the local quality using only the model, whereas the following two approaches consider the ensemble of models. We investigated two different approaches for local quality estimation relying on the structural density information contained in the ensemble of models (QMEANclust_local, selfQMEANlocal).

In the local consensus approach the Cα deviations among the equivalent positions in the models after a sequence-dependent superposition with the program TMscore [52] are analysed in order to derive a quality score. In analogy to the global QMEANclust score, either a subset of all models is used in the distance calculation and the median distance is retrieved, or a weighted mean distance according to the global model quality score is calculated. In this way, segments of more reliable models have a stronger influence on the predicted local score. The model ranking based on QMEANclust is used for model selection and weighting. A weighting according to QMEAN has been also investigated but resulted in a worse performance (data not shown). The statistical potential terms in selfQMEANlocal are trained on the best ranking models of the ensemble. The remaining terms are identical to those in QMEANlocal and the weighting factors are derived using the CASP6 data set.

**Figure 3:**
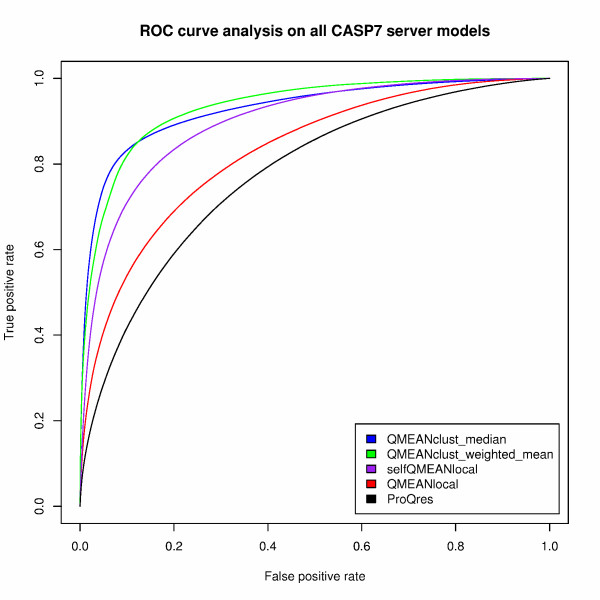
**Receiver operator characteristic (ROC) curves for the different local QMEAN versions and ProQres**. A Cα distance cut-off of 2.5 Å has been used. Two alternative QMEANclust approaches have been tested which combine the local Cα distances using median or weighted mean.

Table 6 shows the evaluation of the local scoring functions using a variety of quality measures covering different performance aspects. The local accuracy of a model is described as the Cα distance between the equivalent residues after superposition of the model and its native structure with TMscore. For each of the 98 CASP7 targets, all residues of all server models are pooled. The target-averaged Pearson's correlation coefficients of the local consensus scoring functions are considerably higher than for the other methods which show almost no linear correlation. Nevertheless, the single model scoring function QMEANlocal shows a strong tendency to discriminate between positions in the models deviating with respect to the native structure from non-deviating positions as reflected by the high average area under curve in the ROC analysis. Two kind of ROC analysis have been performed, one based on all residues of all models per target (average area under curve denoted as ROC_avg _in Table 6) and the other with all models of all targets pooled together (denoted as ROC_all_). The ROC curves of the latter approach (over all 98 targets) are shown in Figure 3. The best performance in estimating the local model quality is achieved by the clustering method QMEANclust_local. The two strategies to calculate the local structural consensus based on the median or weighted average Cα distance among the models result in quite similar curves. The target specific statistical potentials used in selfQMEANlocal perform considerably better than the standard QMEANlocal implementation but do not reach the discrimination power of the consensus methods. In analogy to the global selfQMEAN implementation, the use of target-specific statistical potentials in the local version does not lead to an improved performance as compared to clustering alone. Over all quality measures, QMEANlocal shows a considerably better performance than ProQres.

The last two columns in Table 6 show an analysis of the lowest and highest scoring 10% residues per target according to the corresponding quality score. QMEANlocal shows the best performance in recognising reliable regions as reflected by the best average Cα distance of the lowest scoring 10% residues. As is the case with possibly any other scoring function analysing single models (*i.e*. based on statistical potential terms), QMEANlocal is not able to distinguish regions with high and very high deviation from native. If the model ensemble contains structural redundancy which can be captured by consensus based methods, the local version of QMEANclust is very effective in identifying regions in models which deviate from the structural consensus and regions which are potentially correct. For template-based modelling, correlation coefficients between predicted and calculated local deviation from native were observed as high as 0.95 over the residues of the model ensemble of some CASP7 targets. For the analysis of single models or in the case when the ensemble does not contain useful density information, composite scoring functions such as QMEANlocal may be used. Depending on the modelling situation either one or the other approach may be used to identify incorrect regions in the model which can be subjected to local conformational resampling in a model refinement protocol.

The quality measures described so far all rely on the entire set of residues of all models per target (or over all targets for ROC_all_) and describe the general agreement of predicted and measured local model quality. They do not explicitly analyse whether a method is able to estimate the reliability of different regions *within *a model. Therefore we also analysed for each model the degree of correspondence between predicted and observed local deviation using Kendall's tau rank correlation coefficient. Table 4 reports Kendall's tau averaged over all models per target. The performance of selfQMEANlocal lies between non-clustering and clustering methods.

A ROC curve analysis of the terms contributing to QMEANlocal suggests that the performance is strongly carried by trivial arguments such as solvent accessibility and secondary structure composition (data not shown). Two analogous terms are used both in ProQres and in the SVM approach of Fasnacht *et al*. The performance differences can therefore be partly explained by improved statistical potential terms. The QMEANlocal version presented in this work is only a starting point and a more elaborated approach is needed for combination the terms *e.g*. SVMs or neural networks. Nevertheless, the linear combination of terms used in QMEANlocal performs considerable better than the neural network based ProQres.

## Conclusion

The QMEANclust scoring function described in this work combines the QMEAN composite scoring function which operates on single models with structural density information contained in a model ensemble. We showed that this approach is able to circumvent to some extent the inherent limitations of consensus methods which tend to fail if the best models are not part of the most prominent structural cluster. A statistically significant improvement over other methods relying on structural density information alone is obtained by selecting a subset of models based on the QMEAN score and calculating structural density only with respect to this subset.

The QMEAN scoring function outperforms all non-consensus methods participating at CASP7, both in terms of correlation to GDT_TS and in the task of selecting the best model. The results on the MOULDER test set show that QMEAN has not been specifically optimised for the context of CASP but represents a valuable tool for model selection on more realistic data sets. Compared to the original QMEAN version [33], an all-atom term has been added to the composite scoring function increasing its ability to select good models especially in the template based modelling category. Combining the terms with a more advanced machine learning algorithm may further its performance as model selector for QMEANclust.

At CASP7, consensus based methods have been shown to be superior to methods acting on single models. Nevertheless, none of the participating scoring functions was able at that time to select better models than the best server from Zhang has submitted. The QMEANclust scoring function presented in this work performs significantly better than a naive scoring function always picking Zhang models. The high correlation coefficients obtained for the global and local versions make QMEANclust a good candidate for a refinement protocol. It may be used to enrich the ensemble with good models and to reliably identify deviating regions which then can be subjected to local conformational re-sampling and refinement in a similar way as recently described by the Baker group [59].

The outstanding performance of consensus methods over scoring functions operating on single models at CASP is not observed on the MOULDER test set. The performance of QMEANclust on the more realistic modelling test set highly depends on the composition of the ensemble of models to be analysed. For decoy sets containing many near-native conformations, the performance of the two scoring functions is similar. However, consensus methods will fail on decoy set which include only few near-native protein conformations and do not contain useful consensus information. Performance estimates of consensus methods based on large meta-datasets (e.g. CASP) might overrate their applicability in more realistic modelling situations, and further research is required to investigate the influence of the ensemble composition and the methods used to generate these models.

The two scoring functions QMEAN and QMEANclust are publicly available as part of the QMEAN server [60] under the following address: .

## Methods

### QMEAN and QMEANlocal

The scoring function used in this work for the quality estimation of single models is an extension of the recently published QMEAN composite scoring function [33] consisting of the following five terms: A secondary structure-specific distance-dependent pairwise residue-level potential, a torsion angle potential over three consecutive amino acids, a Cβ solvation potential as well as two terms describing the agreement between predicted and calculated secondary structure and solvent accessibility. See Table 1 for a short description of all terms contributing to QMEAN. Further details about the implementation of the different terms can be found in the original paper.

The new QMEAN version used in this work additionally contains an all-atom interaction potential term in order to be able to capture more details of the models being assessed. The interaction potential is based on all 167 different atom types occurring in proteins and covers distances from 3 to 20 Å (bin size 0.5 Å). It follows the same secondary structure specific implementation as the residue-level potential [33]. Different lower and upper distance cut-offs have been investigated, but these resulted in worse performance on the CASP6 training data set *(data not shown)*.

Optimisation of the weighting factors for the QMEAN composite scoring was performed on the CASP6 training set by using the linear regression module of the R package [61] with the GDT_TS score as target function.

QMEAN = W_torsion _*E_torsion _+ W_solvation _*E_solvation _+ W_pair, residue _*E_pair, residue _+ W_pair, all-atom _*E_pair, all-atom _+ W_SSE agreement _*S_SSE agreement _+ W_ACCagreement _*S_ACCagreement _+ intercept

where:

W_torsion _= -0.00185, W_solvation _= -0.00054, W_pair, residue _= -0.00062, W_pair, all-atom _= -0.00108, W_SSE agreement _= 0.38072, W_ACCagreement _= 0.57997, intercept = -0.28663.

The local scoring function QMEANlocal consists of 8 terms. All terms are calculated over a sliding window of 9 residues and a triangular smoothing weighting scheme has been applied as described elsewhere [16,17]. The same Cβ solvation and residue-level interaction potentials are used as in the global QMEAN scoring function. For the torsion angle potential, a standard implementation with 10 degree angle bins works slightly better than the coarse-grained version over 3 residues used in QMEAN (data not shown). An all-atom interaction potential implementation adapted to local analysis is used covering distances from 0 to 10 Å (step size 0.5 Å). The two agreement terms are adopted and describe the percentage agreement between predicted and measured solvent accessibility and secondary structure within the sliding window. Two trivial features are also used: the average solvent accessibility (weighted by triangular smoothing) and the fraction residues in the 9-residue window with no assigned secondary structure by DSSP [62].

The following weighting factors are used (derived using linear regression in analogy to QMEAN with the Cα distance as target function): W_torsion _= 1.477, W_solvation _= 0.508, W_pair, residue _= 0.164, W_pair, all-atom _= 2.097, W_SSE agreement _= -0.742, W_ACCagreement _= -0.372, W_solvent_accessibility _= 0.051, W_fraction_loop _= 0.666, intercept (with the y-axis) = 1.701.

### QMEANclust and QMEANclust_local

The n*n distance matrix storing all pairwise GDT_TS values between the *n *models is calculated using the program TMscore [52]. Two different approaches to combine QMEAN with structural density information have been investigated: QMEAN is either used to pre-select models before clustering or to weight models during clustering. In the first approach a subset S of models is selected based on the highest QMEAN scores and structural density information is derived by calculating the median GDT_TS score of a given model with respect to all models of the subset S. In order to take into account model completeness, the GDT_TS score between a given model *x *and another model *i *from subset S is multiplied by the fraction of modelled residues *(fm) *of the latter one.



In the second approach the QMEAN score is not used for the pre-selection of models but for weighting each model in the derivation of the structural density score. Distance calculation to models with higher QMEAN score can be considered more reliable and these contain more information than for example a distance to a random model.



In analogy to the analysis of the global deviation between models in QMEANclust, the distance between identical residues after superposition with the software TMscore is used to estimate the local model quality in QMEANclust_local. The Cα distances of all corresponding residues are extracted and stored in a n*n*m matrix (where *n *is the number of models an *m *the length of the complete target sequence).

### selfQMEAN and selfQMEANlocal

For the target-specific versions of QMEAN, the statistical potentials have been derived from all models of a given argets with a QMEANclust Z-score above minus one. Thereby low quality outlier models carrying no information are excluded. The frequency counts (*i.e*. the basis for the different statistical potential terms) are weighted according to the global QMEANclust score. This ensures that structural features of more reliable models have a stronger impact on the resulting potentials. A specific weighting of each interaction according to the local QMEANclust score has also been investigated but resulted in a worse performance. Two approaches for the combination of the statistical potential terms with the agreement terms have been tested: Either the terms are combined directly using the same weighting factors as for QMEAN or Z-scores over all models are built for each term which are then summed up.

### CASP data sets

The training set consists of all models submitted to CASP6. In order to reduce the influence of outliers in the derivation of the weighting factors we applied the following filter. All models which have, for any of the 4 statistical potential terms, a total energy above or below 3 standard deviations, are removed from the training set. This resulted in a final set of 23,925 models.

The CASP7 test set comprises all server models submitted to CASP7. In order to be able to compare our results to those presented in Zhou&Skolnick [35] we only included models of the TS category and skipped AL models. The GDT_TS values for the evaluation were taken directly from the official CASP7 website . All data reported in the tables related to CASP7 represent averages of the 98 targets.

### MOULDER data set

We use the MOULDER test set published in Eramian *et al*. [32] in order to test QMEAN under a more realistic modelling situation. The test set has been originally used to compare the support vector machine based metapredictor SVMod with a variety of existing energy functions. The performance data of all tested scoring functions can be obtained from the Sali Lab  and the comprehensive set of models from the webpage of Marti-Renom . The MOULDER test set from Eramian *et al*. consists of 20 target/template pairs of remotely related homologues. The 20 targets do not share significant structural similarity to each other. For each modelling case a total number of 300 alternative models were generated using MOULDER [7]. We directly used the performance data for all the scoring functions from the publication and re-run the benchmarking including the methods described in this paper.

The performance of a given scoring function in selecting the model closest to the native structure was benchmarked as described in the original paper. From the set of 300 models a random subset of 75 models is selected 2000 times. In each iteration, the models are ranked by the scoring function and the difference (in Ångstrom) between the selected model and the model with the lowest RMSD in the given subset is recorded. Finally, the delta RMSD is reported averaged over the 2000 iterations and 20 targets.

### Benchmarking

The analysis of the statistical significance on the CASP7 set is based on a paired t-test (95% confidence level) and has been carried out in R. The ROC curve analysis has been performed on all residues of all CASP7 server models using the R-package ROCR [63].

In order to evaluate the model quality estimation performance of different local scoring functions a Kendall's tau test has been used to measure the degree of correspondence of RMSD and predicted local score. Kendall's tau has been calculated on a per model basis and compared between the different scoring functions. For this purpose, the Kendall R-Package of A.I. McLeod has been used, accessible over the CRAN website .

## Authors' contributions

PB did the implementation and benchmarking of all QMEAN scoring functions. ST provided intellectual support and supervision during the development of QMEAN and TS for the development of the other three methods. PB drafted the manuscript and TS, ST proofread and extended it. All authors approved the final manuscript.
